# HIV/AIDS-related knowledge and attitudes toward people living with HIV among college students in Xuzhou, Jiangsu Province, China: a cross-sectional survey

**DOI:** 10.3389/fpubh.2024.1398980

**Published:** 2024-10-10

**Authors:** Hualing Li, Qi Wu, Enze Gao, Ying Zhang, Dehui Yin

**Affiliations:** ^1^School of Public Health, Xuzhou Medical University, Xuzhou, Jiangsu, China; ^2^Discipline Inspection Commission, Xuzhou Medical University, Xuzhou, Jiangsu, China

**Keywords:** HIV/AIDS, PLHIV, knowledge, attitude, college students

## Abstract

**Background:**

In the contemporary landscape, college students have emerged as a demographic increasingly vulnerable to AIDS. Recognizing that heightened awareness and progressive attitudes toward HIV are pivotal in its prevention, this study was conceived with the intent to meticulously evaluate the level of HIV understanding amongst college students, gauge their sentiments toward people living with HIV (PLHIV), and scrutinize factors influencing these perceptions.

**Method:**

This study used an anonymous online questionnaire to collect data through a cross-sectional survey. The sample size for the survey was 4,193 students from four colleges and universities in Xuzhou City, Jiangsu Province, China, covering a variety of demographic characteristics. The survey focused on students’ level of knowledge related to HIV and their attitudes toward PLHIV. The study used descriptive statistics to describe the demographic characteristics of the sample, chi-square tests to examine differences within categorical variables, and logistic regression to investigate the associations between knowledge levels and attitudes.

**Results:**

A total of 4,193 university students were surveyed, among whom the majority (96.85%) demonstrated a high level of knowledge related to AIDS. Moreover, 55.52% of the participants reported positive attitudes toward individuals with AIDS. The multiple regression analysis revealed that female students (OR = 0.49, *p* < 0.001), those enrolled in medical programs (OR = 1.56, *p* = 0.014), students of Han Chinese ethnicity (OR = 2.46, *p* = 0.009), and individuals with fewer romantic involvements (OR = 1.57, *p* < 0.001) possess greater HIV/AIDS awareness. Moreover, lower grade levels (OR = 1.12, *p* < 0.001), reduced monthly living costs (OR = 1.14, *p* = 0.014), lack of sexual experience (OR = 0.75, *p* = 0.015), and a higher degree of HIV/AIDS knowledge (OR = 1.617, *p* = 0.007) were positively correlated with supportive attitudes toward PLHIV.

**Conclusion:**

Overall, the awareness rate of college students in Xuzhou City about HIV/AIDS infection awareness is high. However, only about half of the university students have positive attitudes toward PLHIV. In order to ensure that a thorough understanding of HIV/AIDS is matched by positive attitudes, it is important to implement targeted educational measures aimed at bridging the gap between knowledge and attitudes toward HIV/AIDS in order to develop a more informed and empathetic student body.

## 1 Introduction

Over the past few decades, significant strides have been made in understanding and treating the human immunodeficiency virus (HIV), yet the HIV/AIDS epidemic remains a serious global public health issue (1, 2). HIV is a multifaceted disease, with wide-ranging impacts on physical, social, economic, psychological health, and quality of life (3–6). Despite continuous improvements in China’s AIDS prevention policies over the past 40 years, including strengthening public health measures, increasing treatment coverage, promoting preventative knowledge, and continually optimizing interventions for high-risk populations, China remains one of the countries with the largest HIV epidemic in the world (7, 8). Jiangsu Province, one of the most economically developed provinces in China, has recently seen an increase in HIV incidence (9). Xuzhou, located in northern Jiangsu, although having a lower AIDS rate compared to southern Jiangsu, is a crucial transportation hub in the province (10). As a result, a significant number of HIV cases are likely to migrate to other regions (11), thereby presenting a significant and ongoing challenge in prevention and control efforts. The transmission of AIDS among university students represents a substantial health concern (12). In China, newly diagnosed HIV cases among the 15–24 age group, which includes young students, surged from 9,373 in 2010 to 15,790 in 2019 (13). Additionally, young students infected with HIV face significant challenges in their studies and academic pursuits (14). In recent years, HIV infections among students have attracted persistent global attention, focusing on issues such as understanding of HIV/AIDS, risky behaviors, and HIV prevention education (15). One study identified the failure to deliver appropriate HIV/AIDS information as a primary cause of HIV transmission (16). Therefore, understanding the status of HIV infections is paramount for preventing virus transmission, promoting early treatment, and care. A key priority is to heighten awareness of sexually transmitted infections among university students to curb sexual transmission within this age group. To reduce new infections among students, China has adopted measures (17) that combine preventive education, behavioral interventions, and biomedical interventions. However, these measures have not always effectively reached all target groups or comprehensively addressed the need for HIV-related education, which is critical for promoting safe behaviors and reducing stigma. To assess and identify gaps in students’ knowledge and attitudes toward AIDS, the Chinese Center for Disease Control and Prevention conducted a survey in 2021 of 54,052 university students across six cities, revealing significant knowledge and behavioral gaps related to AIDS knowledge and safe sexual practices (18). These findings highlight the need for further research to understand whether students are aware of how HIV is transmitted, who can be a carrier of the disease, and what health behaviors can prevent them from becoming infected or infecting others. Moreover, it is crucial to examine whether students are willing to undergo HIV testing following risky sexual behavior, as this can significantly impact the effectiveness of HIV prevention strategies. Hence, to assess the adequacy of HIV/AIDS education, it is crucial to delve deeply into university students’ understanding of HIV/AIDS and their attitudes toward people living with the virus.

The stigmatization related to HIV manifests as irrational or negative attitudes, behaviors, and judgments toward people living with HIV (PLHIV) or high-risk groups (19, 20). These negative attitudes contribute to prejudice and discriminatory behavior against PLHIV, causing considerable harm (21, 22). A key consequence of such stigma is that it discourages individuals, including students, from getting tested for HIV due to fear of being judged or discriminated against. This avoidance of testing, driven by societal stigma, undermines critical efforts required for managing and preventing the spread of the virus (23, 24). Therefore, studying attitudes toward HIV/AIDS is considered a focal point in HIV/AIDS research (25). It has been established that the negative impact of psychosocial factors on the quality of life among those infected with HIV is greater than that of physiological or medical health determinants (26, 27). Prejudice against PLHIV not only hinders them from seeking help and rebuilding their lives but also deters others from getting tested, which can exacerbate the spread of the virus and worsen living conditions (28). Public acceptance of PLHIV is crucial to reducing HIV-related stigma and discrimination, as well as increasing individuals’ access to HIV testing and other health services (29). Thus, comprehensive educational measures about HIV and AIDS should be undertaken. By raising public knowledge and awareness, efforts can be made to reduce bias and discrimination, and to encourage early HIV testing, which is an essential step in the prevention and control of the disease.

Thus, comprehensive educational measures about HIV and AIDS should be undertaken. By raising public knowledge and awareness, especially among university students, the prevention, education, and control of the disease can be improved.

Despite many efforts made and positive progress achieved worldwide in controlling AIDS, it remains one of the most significant threats to many people’s lives. Prevention is, therefore, the sole approach to tackling this disease. Indeed, it has been observed that students from countries with lower HIV prevalence levels possess a false sense of security, believing they are unlikely to come into contact with PLHIV (30). A retrospective study showed that the dissemination of HIV preventive knowledge could reduce HIV incidence/prevalence to some extent (31). A study on university students connected to knowledge, attitudes, and practices regarding sexually transmitted infections reflects that young people’s attitudes toward sex have become very casual, yet their knowledge remains deficient (32). Moreover, the study shows that prejudice against people living with HIV/AIDS remains prevalent among the Chinese university student population (33). Furthermore, an increase in AIDS knowledge can boost university students’ acceptance of AIDS testing, thus improving detection rates (34). Prejudice against HIV-infected individuals stands as an obstacle in achieving universal access to HIV prevention, treatment, and care plans (35).

In the context of China, cultural and social norms significantly shape public perceptions and responses to health issues such as AIDS. Among Chinese university students, awareness and attitudes toward this disease are deeply influenced by a combination of educational policies, social stigma, and familial expectations. Despite numerous health education programs implemented by the Chinese government, societal stigma and the lack of open communication about sexual health often hinder effective awareness and prevention strategies. The current study was conducted not only to assess the level of HIV-related knowledge among university students but also to examine the stigma toward people living with HIV. It seeks to understand whether students have awareness about the modes of HIV transmission, who might be a carrier of the disease, what preventive health behaviors can protect them from HIV, and whether they would be willing to undergo HIV testing following risky sexual behaviors. Due to the lack of self-motivation for prevention and treatment among university students, there is still much room for improvement in their HIV prevention and health education (12). Building upon previous research, this study aims to comprehensively analyze key factors influencing university students’ cognizance of and attitudes toward AIDS, choosing to focus on the student population in Xuzhou, Jiangsu Province, China. This in-depth examination of specific regional demographics can offer valuable insights pertinent to broader research scopes. The objectives of this study are to: (1) identify the level of HIV/AIDS knowledge among university students, (2) assess their attitudes toward people living with HIV/AIDS (PLHIV), and (3) examine the relationship between knowledge and attitudes. Based on these objectives, the following research hypotheses are proposed: students with higher levels of HIV/AIDS knowledge will demonstrate more positive attitudes toward PLHIV.

## 2 Methods

### 2.1 Survey implementation and characteristics of participants

This cross-sectional study was conducted from September 2023 to November 2023 at four universities in Xuzhou, Jiangsu Province, with a total sample size of 4,261 students. The distribution of participants among the universities was as follows: Xuzhou Medical University accounted for 54.9% of the sample, China University of Mining and Technology represented 16.7%, Jiangsu Normal University contributed 15.1%, and Xuzhou University of Technology made up 13.3%. We used cluster random sampling approach to recruit a sample with diverse majors and from different grades. All data were collected via the WJX (a Chinese online survey platform, https://www.wjx.cn). The electronic survey was designed on this platform and later converted into a QR code for easy access. On-campus students completed the survey by scanning the QR code on their phones, facilitated by their teachers, who guided the students to participate in the study (92.3%). In contrast, students on internships were sent a link to complete the survey independently (7.7%). Prior to completing the questionnaire, students were informed that the results would be used solely for academic research, kept strictly confidential, and were provided with instructions on how to complete the questionnaire. Participants were also informed in advance that the expected time required to complete the questionnaire should not be less than 1 min. The survey was conducted in accordance with the Declaration of Helsinki, an internationally recognized ethical guideline for medical research involving human subjects.

The study initially involved 4,261 participants, but 68 of them were excluded. Of those excluded, 63.2% were due to incomplete or invalid information, and 36.8% were excluded because the time taken to complete the survey was less than 30 s. This left 4,193 participants for the final analysis, with a questionnaire valid response rate of 98.4%. Of these, 1,843 (44.0%) were male and 2,350 (56.0%) were female; 2,338 (55.8%) were medical students, and 1,855 (44.2%) were non-medical students. The distribution by academic year was 44.5% freshmen, 24.6% sophomores, 21.0% juniors, and 9.9% seniors and above. Most participants were Han Chinese (4,062, 96.9%), 1,905 (45.4%) were only children, and 2,611 (62.3%) were from urban areas.

### 2.2 Survey design

In this study, we focus on specific aspects of HIV/AIDS knowledge and attitudes. The scope includes an examination of knowledge regarding transmission routes and prevention methods. Additionally, we analyze attitudes such as stigma and the willingness to interact with people living with HIV (PLHIV). The HIV health education knowledge questionnaire used for data collection in this study was adapted from the “Knowledge of HIV prevention and treatment among adolescents” in the China HIV Sentinel Testing Implementation Program. Questions about attitudes toward PLHIV were developed for this study, these questions were synthesized after extensive literature combing and consultation with professionals in the field of HIV/AIDS. This clarification helps to set clear boundaries for our research, ensuring a comprehensive understanding of the specific elements being investigated.

The first part of the questionnaire collected sociodemographic characteristics of the participants, including grade level, major, gender, nationality, marital status, and monthly cost of living.

The second part used an 8-question questionnaire to assess participants’ knowledge of HIV health education. This indicator was designed by the Chinese National Center for AIDS/STD Control and Prevention and is widely used in national surveillance (36, 37). Scoring was based on one point for each correct answer in each block of HIV health education knowledge. The total score was the total number of correct responses (ranging from 0 to 8). Each correct answer was awarded one point. If the total score reached 6 or above, it indicated that the participant had a good knowledge of HIV prevention (36).

The third section assessed participants’ attitudes toward PLHIV. For each question regarding attitudes toward PLHIV, a negative response was scored as 0, and a positive response was scored as 1. With a total of seven questions, participants who scored 4 or above were considered to have a positive attitude toward PLHIV.

### 2.3 Statistical analysis

Count data were expressed as *N* (%). Multifactorial logistic regression was used to assess the possible factors (gender, grade, major, ethnicity, being an only child or not, native place, monthly living expenses, sexual orientation, number of love affairs, and whether or not they have ever had sexual intercourse) associated with the level of knowledge related to HIV/AIDS and attitudes toward PLHIV. The corresponding results are expressed as odds ratios (OR) and 95% confidence intervals (CI). *p*-values <0.05 were considered statistically significant. All analyses and plots were performed using SPSS 26.0 and GraphPad Prism version 10.0.

## 3 Results

### 3.1 Sociodemographic characteristics of participants (*N* = 4,193)

In this survey, there were 1843 (44.0%) males and 2,350 (56.0%) females. There were 2,338 (55.8%) medical students and 1,855 (44.2%) non-medical students. Students from freshman to senior year and above accounted for 44.5, 24.6, 21.0, and 9.9%, respectively. The participants were mainly Han Chinese (4,062, 96.9%). There were 1,905 only children (45.4%), and the urban population was larger (2,611, 62.3%). The largest proportion of students, 70.3%, had a monthly living expense of 1,000–2,000 yuan. Students who were heterosexual (3,432, 81.9%), had never been in a relationship (3,108, 74.1%), and had never had sex (3,805, 90.7%) made up the majority.

### 3.2 HIV/AIDS related knowledge

Overall, 96.9% of the participants had a high level of HIV/AIDS related knowledge. Of the eight HIV/AIDS related knowledge questions, six were correct at 90% or above. The three questions with the highest number of correct answers were that one should take the initiative to seek HIV testing and counseling after engaging in high-risk behaviors (98.7%), that the use of new types of drugs increases the risk of contracting HIV (97.3%), and that adhering to the correct use of condoms can reduce the risk of contracting and transmitting HIV (96.3%). The two questions with less than 90% correct were that AIDS is an incurable and serious infectious disease (82.4%) and that the main mode of transmission of AIDS among young students in China is male homosexual sex (81.2%) (Table 1).

**Table 1:** Cognitive levels on the HIV/AIDS-related knowledge among the college students in Xuzhou, Jiangsu Province, China (*n* = 4,193).

Questions	Correct number (%)
1. Is AIDS an incurable and serious infectious disease?	3,457(82.4)
2. At present, the prevalence of AIDS among young students in our country is increasing rapidly. The main mode of transmission is male–male sexual behaviors.	3,405(81.2)
3. Can a person with HIV be distinguished by his appearance?	3,821(91.1)
4. Can a person get HIV from daily life and contact?	3,844(91.7)
5.Can the consistent use of condoms reduce HIV/AIDS transmission?	4,036(96.3)
6. Will the use of new drugs (such as methamphetamine, ecstasy, K powder, etc.) increase the risk of acquiring HIV?	4,081(97.3)
7. Should HIV testing and counseling be actively sought after high-risk behaviors (e.g., needle and implement sharing/unsafe sex among drug users)?	4,138(98.7)
8.The rights and interests of PLHIV, such as marriage/employment/school enrollment, are protected by our laws?	3,952(94.3)

### 3.3 Attitudes toward PLHIV

Overall, 55.5% of the participants had a positive attitude toward PLHIV. Specifically, more than half of the respondents were not willing to have contact with PLHIV (59.8%), but 53.8% of the students were willing to continue to socialize with classmates or friends infected with HIV. 43.5% of the students were uncomfortable shaking hands with a person infected with HIV. 58.9% were uncomfortable eating at the same table as a person infected with HIV. 60.0% were not willing to buy food from a person infected with HIV. 88.2% disagreed with the statement that “people with HIV should be banned from working.” 88.2% of the students disagreed with the statement “AIDS patients should be prohibited from working.” 84.4% of the students disagreed with the statement “AIDS is a punishment for bad behavior” (Table 2).

**Table 2:** Participants’ attitudes toward PLHIV (*n* = 4,193).

Questions	Participants (%)
Q1. Are you willing to have contact with a person with AIDS?	
Yes	1,687(40.2)
No	2,506(59.8)
Q2. Would you continue to socialize with your classmate or friend if he/she was infected with HIV?	
Yes	2,257(53.8)
No	1936(46.2)
Q3. Are you uncomfortable shaking hands with someone living with HIV?	
Yes	1825(43.5)
No	2,368(56.5)
Q4. Are you uncomfortable eating at the same table as someone living with HIV?	
Yes	2,470(58.9)
No	1723(41.1)
Q5. Would you be willing to buy food from a salesperson if he/she had AIDS?	
Yes	1,679(40.0)
No	2,514(60.0)
Q6. Do you agree with the statement “AIDS patients should be prohibited from working”?	
Yes	494(11.8)
No	3,699(88.2)
Q7. Do you agree with the statement “AIDS is a punishment for bad behavior”?	
Yes	656(15.6)
No	3,537(84.4)

### 3.4 Analysis of factors influencing the knowledge of college students about AIDS

**Figure 1:**
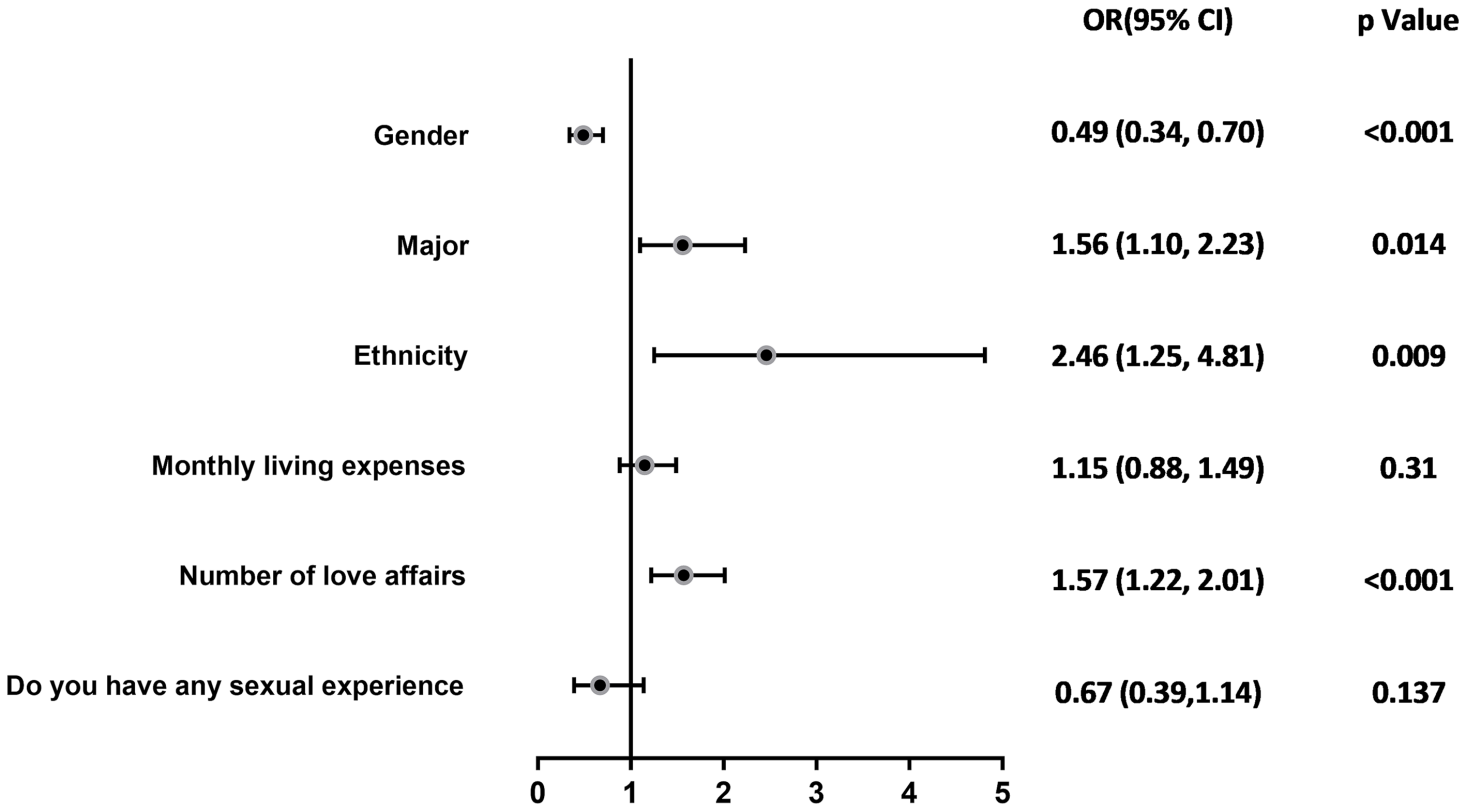
Logistic regression analysis of college students’ knowledge of HIV/AIDS–related knowledge.

The results of the univariate analysis of the qualification of knowledge about AIDS were grouped and related to multiple variables, which showed that gender (*χ*^2^ = 13.977, *p* < 0.001), major (*χ*^2^ = 5.867, *p* = 0.015), ethnicity (*χ*^2^ = 14.060, *p* < 0.001), monthly living expenses (*χ*^2^ = 54.880, *p* < 0.001), number of love affairs (*χ*^2^ = 130.825), and whether having sex experience (*χ*^2^ = 29.465, *p* < 0.001) were the most important variables in the analysis of the results of the univariate analysis of the study. The differences were statistically significant (see Table 3). The above factors were included in the multivariate regression analyses as independent variables, and the qualification of knowledge about AIDS was included in the multifactorial regression analyses as a dependent variable, and the results showed that the differences were statistically significant for gender (OR = 0.49, *p* < 0.001), major (OR = 1.56, *p* = 0.014), ethnicity (OR = 2.46, *p* = 0.009), and number of love affairs (OR = 1.57, *p* < 0.001) are the influencing factors affecting the rate of college students’ knowledge about AIDS, as shown in Table 4 and Figure 1. Female college students, medical students, Han college students, and college students with fewer number of love affairs have a better knowledge of AIDS-related knowledge.

**Table 3:** Single-factor analysis of college students’ knowledge of HIV/AIDS–related issues.

Variables	*N*	Score ≥ 6	Score < 6	*χ* ^2^	P
Gender				13.977	<0.001
Male	1,843(44.0)	1764(95.7)	79(4.3)		
Female	2,350(56.0)	2,297(97.7)	53(2.3)		
Major				5.867	0.015
Medical	2,338(55.8)	2,278(97.4)	60(2.6)		
Non-medical	1,855(44.2)	1,783(96.1)	72(3.9)		
Grade				3.769	0.284
Freshman	1,866(44.5)	1804(96.7)	62(3.3)		
Sophomore	1,033(24.6)	996(96.4)	37(3.6)		
Junior	881(21.0)	855 (97.0)	26(3.0)		
Senior or more	413(9.9)	406(98.3)	7(1.7)		
Ethnicity				14.060	<0.001
Han	4,062(96.9)	3,942(97.0)	120(3.0)		
Minority	131(3.1)	119(90.8)	12(9.2)		
Only child or not				0.798	0.372
Yes	1,905(45.4)	1,840(96.6)	65(3.4)		
No	2,288(54.6)	2,221(55.8)	67(2.9)		
Native place				0.048	0.827
Urban	2,611(62.3)	2,530(96.9)	81(3.1)		
Rural	1,582(37.7)	1,531(96.8)	51(3.2)		
Monthly living expenses				54.880	<0.001
≤1,000 CNY	194(4.6)	178(91.8)	16(8.2)		
1,000–2,000 CNY	2,949(70.3)	2,876(97.5)	73(2.5)		
2,000–3,000 CNY	902(21.5)	876(97.1)	26(2.9)		
≥3,000 CNY	148(3.5)	131(88.5)	17(11.5)		
Your sexual orientation				2.893	0.408
Heterosexual	3,432(81.9)	3,330(97.0)	102(3.0)		
Homosexual	85(2.0)	82(96.5)	3 (3.5)		
Bisexual	248(5.9)	236(95.2)	12(4.8)		
Not sure	428(10.2)	413(96.5)	15(3.5)		
Number of love affairs				130.825	<0.001
None	3,108(74.1)	3,019(97.1)	89(2.9)		
1–2 times	954(22.8)	934(97.9)	20(2.1)		
3–5 times	51(1.2)	48(94.1)	3(5.9)		
More than 5 times	80(1.9)	60(75.0)	20(25.0)		
Do you have any sexual experience?				29.465	<0.001
Yes	388(9.3)	358(92.3)	30(7.7)		
No	3,805(90.7)	3,703(97.3)	102(2.7)		

**Table 4:** Multifactorial analysis of college students’ knowledge of HIV/AIDS–related knowledge.

Variables	β	SE	Wasd *χ*^2^	OR (95% CI)	P
Gender	−0.718	0.186	14.905	0.49 (0.34, 0.70)	<0.001
Major	0.445	0.181	6.070	1.56 (1.10, 2.23)	0.014
Ethnicity	0.898	0.343	6.858	2.46 (1.25, 4.81)	0.009
Monthly living expenses	0.137	0.135	1.030	1.15 (0.88, 1.49)	0.310
Number of love affairs	0.451	0.127	12.550	1.57 (1.22, 2.01)	<0.001
Do you have any sexual experience?	−0.403	0.271	2.208	0.67 (0.39,1.14)	0.137

### 3.5 Analysis of factors influencing college students’ attitudes toward PLHIV

**Figure 2:**
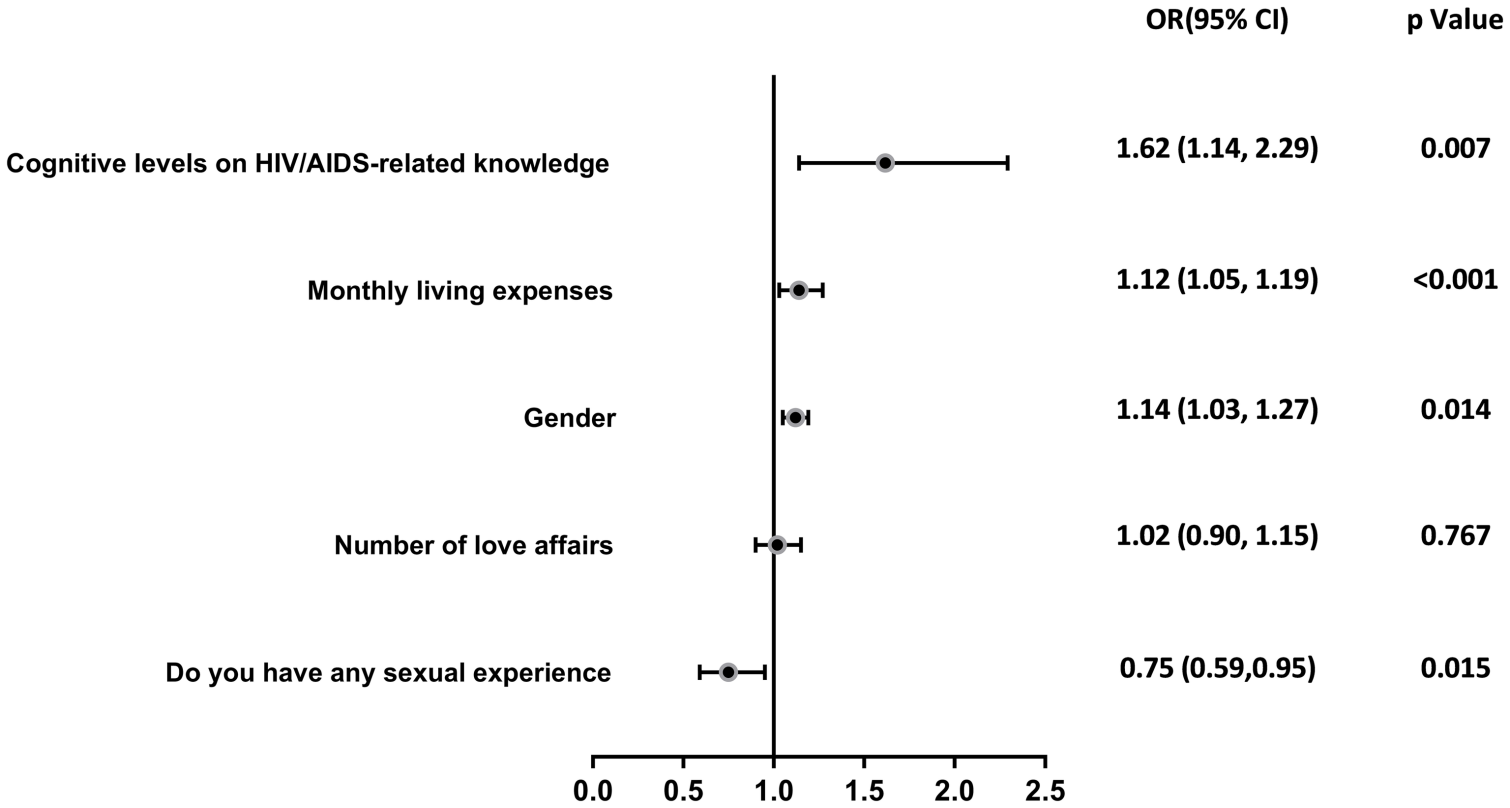
Logistic regression analysis of college students’ attitudes toward PLHIV.

The study participants’ attitudes toward HIV were grouped and analyzed with several variables in a univariate analysis, and the results showed that grade (*χ*^2^ = 21.775, *p* < 0.001), monthly living expenses (*χ*^2^ = 11.715, *p* = 0.008), number of love affairs (*χ*^2^ = 10.172, *p* = 0.017), whether having sex experience (*χ*^2^ = 14.430, *P* < 0.001), and cognitive levels on HIV/AIDS-related knowledge (*χ*^2^ = 7.403, *p* = 0.007) were the most important factors. The differences were statistically significant, as shown in Table 5. The inclusion of the above factors as independent variables and attitudes toward PLHIV as the dependent variable in multifactor regression analyses showed that grades (OR = 1.12, *p* < 0.001), monthly living expenses (OR = 1.14, *p* = 0.014), whether having sex experience (OR = 0.75, *p* = 0.015), and cognitive levels on HIV/AIDS-related knowledge (OR = 1.617, *p* = 0.007) are the influencing factors affecting the college students’ attitudes toward PLHIV (see Table 6 and Figure 2). College students who were in lower grades, had low cost of living, and had not had any sexual experience had positive attitudes toward PLHIV.

**Table 5:** One-way analysis of college students’ attitudes toward PLHIV.

Variables	*N*	Positive attitude	Negative attitude	*χ* ^2^	P
Gender				0.020	0.888
Male	1,843(44.0)	1,021(55.4)	822(44.6)		
Female	2,350(56.0)	1,307(55.6)	1,043(44.4)		
Major				0.137	0.711
Medical	2,338(55.8)	1,304(55.8)	1,034(44.2)		
Non-medical	1,855(44.2)	1,024 (55.2)	831(44.8)		
Grade				21.775	<0.001
Freshman	1,866(44.5)	1,107(59.3)	759(40.7)		
Sophomore	1,033(24.6)	558(54.0)	475(46.0)		
Junior	881(21.0)	447(50.7)	434(49.3)		
Senior or more	413(9.9)	216(52.3)	197(47.7)		
Ethnicity				0.051	0.821
Han	4,062(96.9)	2,254(55.5)	1808(44.5)		
Minority	131(3.1)	74(56.5)	57(43.5)		
Only child or not				0.156	0.693
Yes	1,905(45.4)	1,064(55.9)	841(44.1)		
No	2,288(54.6)	1,264(55.2)	1,024(44.8)		
Native place				3.144	0.076
Urban	2,611(62.3)	1,422(54.5)	1,189(45.5)		
Rural	1,582(37.7)	906(57.3)	676(42.7)		
Monthly living expenses				11.715	0.008
≤1,000 CNY	194(4.6)	105(54.1)	89(45.9)		
1,000–2,000 CNY	2,949(70.3)	1,678(56.9)	1,271(43.1)		
2,000–3,000 CNY	902(21.5)	479(53.1)	423(46.9)		
≥3,000 CNY	148(3.5)	66(44.6)	82(55.4)		
Your sexual orientation				5.988	0.112
Heterosexual	3,432(81.9)	1880(54.8)	1,552(45.2)		
Homosexual	85(2.0)	53(62.4)	32 (37.6)		
Bisexual	248(5.9)	152(61.3)	96(38.7)		
Not sure	428(10.2)	243(56.8)	185(43.2)		
Number of love affairs				10.172	0.017
None	3,108(74.1)	1768(56.9)	1,340(43.1)		
1–2 times	954(22.8)	493(51.7)	461(48.3)		
3–5 times	51(1.2)	29(56.9)	22(43.1)		
More than 5 times	80(1.9)	38(47.5)	42(52.5)		
Do you have any sexual experience?				14.430	<0.001
Yes	388(9.3)	180(46.4)	208(53.6)		
No	3,805(90.7)	2,148(56.5)	1,657(43.5)		
Cognitive levels on HIV/AIDS-related knowledge				7.403	0.007
Score ≥ 6	4,061(96.9)	2,270(55.9)	1791(44.1)		
Score < 6	132(3.1)	58(43.9)	74(56.1)		

**Table 6:** Multifactorial analysis of college students’ attitudes toward PLHIV.

Variables	β	SE	Wasd*χ*^2^	OR (95% CI)	P
Grade	0.114	0.031	13.009	1.12 (1.05, 1.19)	<0.001
Monthly living expenses	0.132	0.054	6.013	1.14 (1.03, 1.27)	0.014
Number of love affairs	0.018	0.060	0.088	1.02 (0.90, 1.15)	0.767
Do you have any sexual experience?	−0.293	0.121	5.900	0.75 (0.59,0.95)	0.015
Cognitive levels on HIV/AIDS-related knowledge	0.481	0.178	7.275	1.62 (1.14,2.29)	0.007

## 4 Discussion

The study results reflect the demographic characteristics, knowledge of AIDS, and attitudes toward PLHIV among college students in Xuzhou City, Jiangsu Province, China. Notably, a high proportion of participants (96.9%) demonstrated awareness of AIDS-related knowledge (Table 1), exceeding reports from Eastern China and Henan Province (90.9, 80.8%) (38, 39). This suggests that educational efforts regarding AIDS within the university setting have been successfully disseminated and embraced. However, the high proportion of medical students in our sample could have skewed these results. A substantial 55.8% of our sample were medical students, who are likely more familiar with health-related topics compared to the general population. This specific composition of our sample could limit the applicability of our findings to broader populations. Medical schools include AIDS education in their curriculum. Previous research has shown that teaching methods incorporating experiential learning, small group discussions, affective components, and positive attitude role models can effectively change students’ attitudes (40, 41). Therefore, it is necessary to integrate these educational measures into non-medical curricula. Additionally, the accuracy for question Q2 “At present, the prevalence of AIDS among young students in our country is increasing rapidly. The main mode of transmission is male–male sexual behaviors” was the lowest at 81.2%, similarly, a study in 2021 involving 54,052 college students showed an even lower accuracy of 66.8% (18), indicating that the knowledge that HIV/AIDS is mainly transmitted through homosexual contact among Chinese young students has not been fully popularized.

The research revealed key factors influencing college students’ understanding and perception of HIV/AIDS, including gender, field of study, ethnic background, and dating experience. Specifically, female students (OR = 0.49, 95%CI: 0.34–0.70; *p* < 0.001), medical students (OR = 1.56, 95%CI: 1.10–2.23; *p* = 0.014), Han Chinese students (OR = 2.46, 95%CI: 1.25–4.81; *p* = 0.009), and students with less dating experience (OR = 1.57, 95%CI: 1.22–2.01; *p* < 0.001) demonstrated higher levels of awareness about AIDS, which might be attributed to targeted educational programs, cultural backgrounds, and personal behaviors. Considering that medical students play a role in health promotion and in the prevention, education, management, and treatment of diseases at personal, family, and community levels, this group could also help in disseminating AIDS-related knowledge on a societal level (42). In terms of gender, the research indicated that female students’ level of knowledge about AIDS was higher than that of male students, which might be due to women’s greater concern for health matters and more proactive approach in communication and information acquisition (43, 44). Current studies have shown that male students comprise the majority of HIV/AIDS cases in universities (45), suggesting a possible disconnect between knowledge and behavior and the need for enhanced AIDS prevention education among male student populations to reduce risk of infection (46). The academic major of university students significantly impacts their awareness of AIDS-related knowledge. The results indicate that medical students exhibit a higher level of awareness. This is likely due to the extensive coverage of diseases, prevention, and treatment within medical education, where medical students are exposed to a broader range of information pertinent to the medical field (34), resulting in a deeper understanding of AIDS, highlighting the importance of professional training in improving specific health knowledge. The effect of ethnic background showed that Han Chinese students had a higher level of AIDS knowledge compared to ethnic minorities, possibly due to linguistic and access challenges faced by minority groups (47). This underscores the necessity for inclusive educational strategies that address language and cultural barriers to enhance information accessibility and equity in health education. Lastly, dating experience, as a psychosocial factor affecting AIDS knowledge, revealed that students with less dating experience had a better understanding of AIDS. This may suggest that these students are more receptive to formal educational messages rather than personal anecdotes, indicating the importance of authoritative sources in health education. This aligns with the study’s objective of identifying key factors influencing HIV/AIDS knowledge.

AIDS education programs should consider the diversity of student populations and a range of social variables to effectively increase college students’ knowledge and awareness of AIDS. Customizing educational content according to the specific needs of different groups can enhance the acceptability and impact of educational initiatives, promoting the dissemination of health information and the progress of AIDS prevention and control efforts.

Our research found that 55.52% of college students have a positive attitude toward people living with AIDS, while a European study reported that 54.67% (556/1017) of students have a positive attitude toward individuals infected with HIV (38). This fulfills the second objective. However, a study focused exclusively on medical students showed that two-thirds of them hold positive attitudes toward people living with AIDS, likely due to their professional training (48). Notably, lower-grade students were found to have more positive attitudes compared to higher-grade students (OR = 1.12, 95%CI: 1.05–1.19; *p* < 0.001), suggesting that younger, less academically experienced students may be more open and empathetic. This could imply that interventions to foster positive attitudes toward HIV/AIDS should ideally be implemented early in students’ academic journeys. Furthermore, students with lower living expenses showed better attitudes toward HIV/AIDS patients (OR = 1.14, 95%CI: 1.03–1.27; *p* = 0.014), potentially reflecting their empathy with marginalized groups in society. This implies that economic factors and social standing may impact sympathy and support for individuals infected with the AIDS virus (29, 49). To further these efforts, targeted interventions should focus on empathy-building workshops, peer-led discussions, and educational programs that begin early in students’ academic careers. Emphasizing the personal stories and lived experiences of people living with HIV/AIDS can foster a deeper understanding and reduce stigma. Additionally, addressing economic and social disparities within the student population can enhance empathy and support for marginalized groups.

However, our study found that students with a comprehensive understanding of HIV/AIDS did not necessarily exhibit positive attitudes (OR = 1.62, 95%CI: 1.14–2.29; *p* = 0.007). This fulfills the third objective of assessing attitudes, showing that while knowledge is high, positive attitudes are not as widespread. This contradicts the initial hypothesis, as knowledge alone did not ensure more favorable attitudes. This is inconsistent with the findings of previous study (50), a finding that can be understood in the context of stigma theory, which suggests that despite possessing sufficient knowledge, individuals may still hold negative attitudes due to the deep-rooted social stigma associated with HIV. Even with a high level of knowledge, stigma persists and influences behavior and attitudes toward people living with HIV (PLHIV). In fact, students with higher levels of knowledge were more likely to have negative attitudes toward PLHIV. This paradox highlights the need for comprehensive education strategies that include empathetic and ethical components to effectively change perceptions and behaviors. Personal experience is also a key influencing factor, as students with a history of sexual activity showed a significantly higher level of acceptance (OR = 0.75, 95%CI: 0.59–0.95; *p* = 0.015). This suggests that personal experiences play a crucial role in eliminating fears related to HIV/AIDS. For students with a history of sexual activity, focus on empathy-building workshops and peer discussions to reinforce positive attitudes and promote safe sex practices and regular HIV testing.

These findings indicate that simple knowledge transmission is limited, and personal experience is essential in shaping attitudes. Therefore, educational programs should consider the diversity and social variables of student populations and adopt a multifaceted approach to enhance awareness of AIDS and support for patients (41). These include sharing personal stories from individuals living with HIV to humanize their experiences, organizing interactive workshops to enhance students’ empathy, and facilitating discussions on ethical principles like respect, confidentiality, and non-discrimination.

## 5 Conclusion

The study indicates that university students in Xuzhou City possess a relatively high awareness of HIV-related subjects. However, their attitudes toward PLHIV could be improved. It is important to recognize that deep knowledge and positive attitudes should ideally complement one another. To this end, it would be beneficial to develop thoughtful prevention policies that aim to close the gaps in knowledge and attitudes related to HIV/AIDS issues, thus nurturing a more empathetic and informed outlook among students toward PLHIV. Implementing similar educational strategies worldwide could lead to a more compassionate and well-informed global community, ultimately enhancing the effectiveness of HIV/AIDS prevention efforts everywhere.

### 5.1 Limitations

This study has several limitations. First, as a cross-sectional study, it can only show associations, not causal relationships. Second, response bias may exist due to the self-administered online questionnaire, which may not fully reflect true attitudes or knowledge. Third, the sample was limited to universities in Xuzhou City, Jiangsu Province, which restricts the generalizability of the findings. Fourth, the survey focused on cognitive rather than behavioral aspects, asking participants what they should do rather than what they actually do; future studies should address this by including actual behavior-focused questions. Fifth, further research could examine whether individuals with higher HIV knowledge are more inclined to seek HIV testing after unsafe sex, using HIV knowledge as a predictor and testing intention as the outcome. Sixth, the reliance on self-reported attitudes may not accurately reflect actual behaviors due to social desirability bias. Lastly, while demographic data is included, a more detailed breakdown is needed for assessing representativeness. Future research should involve longitudinal studies and more diverse regions for broader applicability.

## Data Availability

The raw data supporting the conclusions of this article will be made available by the authors, without undue reservation.
